# A ‘Learning conversation’ as a style of feedback

**DOI:** 10.15694/mep.2017.000156

**Published:** 2017-09-12

**Authors:** Elizabeth M. Norris, Ian Bullock

**Affiliations:** 1 Resuscitation Council UK; 2 Royal College Physicians

**Keywords:** Feedback, Active listening, Reflective practice

## Abstract

This article was migrated. The article was marked as recommended.

This paper explores the history behind the introduction and use of a ‘learning conversation’ as a mechanism of providing effective feedback to students on resuscitation courses. The authors hope the use of this style of feedback may useful in many other contexts to provide authentic feedback. The central aim of a learning conversation is to promote and support greater self-awareness of the individual student in order to develop competence and team leadership through critical inquiry ( Harri-Augstein & Thomas 1991). Learning, particularly in the context of resuscitation is demonstrated by the utilisation of “planned experience which brings about a change of behaviour” and the process is facilitated by feedback whether during skills teaching, in workshops or in simulation. The learning conversation uses empathic, active respectful listening and discussion shared between a small group of participants, facilitated by an instructor to ensure that key learning emerges from this process duly informing future practice and behaviours. A mnemonic has been developed to facilitate acquisition of the skills involved in this feedback. Faculty also require feedback to fully develop their feedback skills but once established the process makes both faculty and students share their feelings, frustrations and learning in a very positive learning climate. The learning conversation has been in use in resuscitation courses for almost six years and the authors feel there is a wealth of literature available to support this approach which can be usefully applied to facilitate learning in many small group teaching settings and the process of delivering a Learning conversation is detailed within the paper.

## Introduction

The learning conversation was developed initially by two educators for the Advanced Life Support group: Dr Davis and Dr Denning six years ago as a mechanism for providing effective feedback to students on resuscitation courses and evidence to support its introduction initially was published in 2016 (Chapter 8. Pocket guide to teaching for Clinical instructors). Adults are by nature independent learners and generally choose to learn what they feel is relevant to them, they construct their learning on what they read, see and do and the degree of retention depends on their perception of what is useful. Such self-organised learning creates meaning from experience (actual and simulated) Knowles MS et al 2005 and feedback is fundamental to reflective practice and the supervision of learners. There has been a paradigm shift in recent years such that feedback is thought to be as important as simulation practice itself in terms of learning, it not only encourages reflection but offers processes to improve. ( Issenberg et al 2005).

### History

The desire to fully embrace androgogy has led to a change in the teaching process over the last 30 years, resulting in essentially 3 teaching styles:

A Instructor-focused style where transmission of knowledge and skills is delivered in a passive way, student facilitated learning concerning acquisition of knowledge and skills with the help of the instructor and finally learner focused where the emphasis lies on differentiating teaching to each individual’s needs. The lattermost requires more active participation by the students in terms of reflection and critical thinking ( Calkins et al 2012).

In a similar way feedback has evolved to match this understanding. The Pendleton model, one of the most frequently used is a conversational model relating to performance consisting of 4 steps:

• the student states what was good about his or her performance
• the instructor states areas where they agree
• the learner states what was poor or could have been done differently
• the instructor states what could be improved ( Pendleton D et al 2003)

This model has existed in the UK since the early 1980’s and was the standard training tool used for general practice trainees and has been used in resuscitation teaching for many years. One of the difficulties with Pendleton lies in the instructor providing the structure and ‘leading’ the direction of the feedback process and at times this style of feedback can feel very artificial. Considerable time is often used in positive reinforcement and the learner’s own perspective/ agenda may either never really emerge or is reached late in the feedback leaving, little time to even remember or explore key issues. It can be difficult for students to try and reflect on both sides of an action in such a structured way, especially when reflection on action is unable to provide adequate time to do so ( Schon 1987).

Since teaching methods have changed to more of a facilitative approach, feedback has similarly evolved using a reflective model where students are able to discuss their performance in relation to achieving the goals of a teaching session. Silverman et al 1997 described a different model - ‘the SET-GO’ approach which forms the basis for teaching clinical communication skills to many doctors today in the UK and North America. This approach consists of the following elements:

• The feedback is descriptive of actions rather than evaluative
• The feedback is specific in order to promote change in behaviour
• The feedback is personalised so learner centred• The interpretation of the feedback is checked with the learner
• The feedback is limited in quantity to an amount which can be usefully applied• Inquiry checks observations and offers potential solutions
• Suggestions are made rather than prescriptive comments

This communication process is inclusive of 5 essential skills ( Silverman 1997).

1. Dialogue must be interactive with an emphasis on interdependency
2. The communication should be clear, the aim is to reduce uncertainty
3. Good communication requires planning and thinking in terms of driving the conversation towards the desired outcome
4. The dialogue should be dynamic, involving both parties
5. The communication should follow a helical model as suggested by the spiral curriculum ( Harden & Stamper 1999).

This model is similar to the model now used in high fidelity simulation ( Rudolph et al 2007). The goal of feedback on resuscitation courses is to allow the candidate to reflect, explain actions, and in the context of teamwork to use the peer support and power of ‘group-think’ to challenge thinking and offer new ideas in order to improve both personal and team performance in the future. Instructors should reinforce a supportive atmosphere throughout resuscitation courses because a safe atmosphere is essential to deliver such thought provoking, authentic and at times challenging feedback whilst maintaining the self esteem of the individual. The relationships between instructors and students continue to develop through the course to reach mutual trust, showing sensitivity to the stress of the learner and this buffers candidate anxiety. This relationship is vitally important to overcome candidate reluctance to receive feedback which may otherwise form a barrier to fruitful dialogue. Current teaching emphasises that feedback should be collaborative (with the student) to share their understanding with their team and scaffold their learning, whilst simultaneously guiding the reflection towards clear learning outcomes in a teaching session. This forms the basis of a learning conversation. A relaxed atmosphere helps the learner to absorb their feedback and maximise their learning and requires a skilled instructor ( Murdoch-Eaton & Bowen 2017).

## The Skills Involved

**Figure 1.**
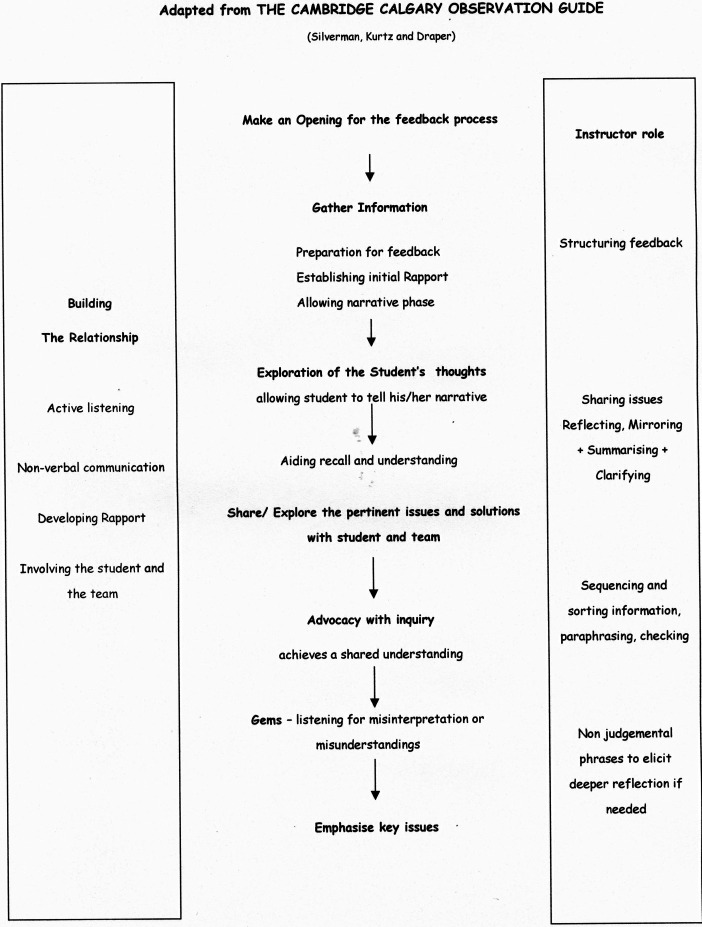
A Learning Conversation

Faculty do need training in this or indeed any approach to feedback ( Brucker et al 1999). Dr Denning introduced the method first to the RC-UK educator group and the knowledge was disseminated to all instructors across all their courses with the aid of handouts and ongoing feedback which continues today. The learning conversation offers opportunity to revisit the clinical experience (the simulation or skill station etc), to explore and link with prior knowledge, promoting higher levels of activity within the cognitive domain leading to deeper learning and informing future practice and behaviours. This organised ‘talk-back” is a discussion to identify key points for discussion, to correct errors, questioning and promoting effective learning. This framework fits well with the Kolb experiential learning cycle, where individuals experience an event, reflect, conceptualise their actions and then have opportunity for further practice allowing change in behaviour (moving between the ‘concrete’ to the ‘abstract’ in order to then inform future practice). The facilitative instructor providing feedback uses the structure of the learning conversation to create a debriefing collaborative dialogue requiring active listening, acute observation followed by critical and reflective analysis of the student’s own performance retrospectively, and in practice it is the joint exploration of erroneous thinking or practice where most dynamic (leading to future change) learning takes place ( Silverman et al 1997). It is a referent dialogue, using the candidate’s understanding of themselves and others (their team) to exchange strategies, purpose and outcomes. Many of the communication skills used within the doctor-patient consultation are valid in the context of this style of feedback. **See Figure 1**

Unlike high fidelity simulation (HFS) training the time allowed for debrief on Advanced life support courses is approximately five minutes within a fifteen minute scenario simulation. Generally in HFS the times allotted are at least the same if not longer for debrief. HFS has shown the ideal is for individuals and teams to debrief themselves as far as possible and at the same time meet their learning objectives.

### Active listening

Active listening is about hearing what the other person is communicating, both verbally and non verbally and responding to what has been heard ( Emerson 2006). Dobkin & LaLiberté 2014 illustrate how being ‘mindful’ as a clinical teacher can help acquire the skill of active listening. The instructor must be enthusiastic and focused because active listening means being aware of one’s own emotions, body language and language content as well in addition to those of the candidate.; Boudreau et al 2009 describe active listening as a “triadic process involving the speaker, the utterance and the listener”, there may be cultural and or maturational differences which need to be considered. Active listening is non-judgemental and allows the student’s view to be heard and understood and it therefore depends a great deal on the attitude of the mentor/facilitator. Thoughts, feelings and irritations concerning students can interfere with our ability to listen, show empathy and give good direction to students. The learner’s feelings, opinions and person should be valued with respect for their fears and hesitancy and this will increase the confidence and trust of both the individual and team members to share problems.

### Opening remarks

The process itself can be started with an opening remark to encourage the candidate (team leader) to share their immediate thoughts relating to their performance. By design, it encourages joint exploration between facilitator, candidate and the team. Alternatively, a pause will allow time for the candidate to gather their own thoughts prior to joint exploration in the feedback process. The instructor emphasis here is to promote self and group reflection, dwelling on uncertainties, accepting ideas and suggestions in a collaborative approach preparing the candidate(s) for future learning/practice. The facilitator builds the relationship with careful verbal and non verbal communication. This exploration requires patience because student(s) need time to reflect and then tell their narrative. Langewitz 2002 showed that doctors interrupt their patients after just 22 seconds and take the lead. If patients are allowed to tell their narrative with simple non verbal facilitation “hmm-hmm” and nodding then the average spontaneous talking time was 92 seconds with a median of 59 seconds. Students must be allowed this same opportunity and time to self reflect. This narrative is not a ‘list’ of what happened, it is the first reflection of the candidate’s emotional reaction to events and most students immediately identify useful issues to explore. A ‘list’ of events is neither insightful nor reflective and should be discouraged.

### Creating the mind set for inquiry

Rogers 1994 says that by posing exploratory questions within a responsive, empathic climate one is helping students develop self directed learning, which helps to foster continuity of learning. Feedback to a student is an opportunity to build or destroy confidence, it is a communication skill that can be both taught and learned. Facilitators want learners to participate fully and freely in a learning conversation so that their knowledge and perspective can be explored and modified to achieve learning goals, and perhaps most importantly point them forward to the next similar experience. Adult learners will then reconstruct their learning needs and find new strategies to achieve those outcomes. Rogers also proposes adults will resist behaviour change if they feel threatened; reorganisation of thoughts and reframing occurs better when people are relaxed. Harden and Laidlow 2013 talked about four key principles to help the feedback process: FAIR - provide *Feedback*, engage the student to create *Active* learning, *Individualise* feedback and make learning *Relevant.*

### Exploration

Joint exploration seeks to bring the candidate’s knowledge to the surface and find explanation for thinking and actions. It may be both insightful and innovative for the whole team generating new ideas; the candidate and the team need to be ‘open’ to accept remedial help if necessary. The empathic nature of this approach is attentive to the individuals’ self-esteem and confidence, seeking to build both in a positive way. Evidence supports learning viewed as a spiral process of revisiting and extending knowledge and skills, providing scaffolding and reinforcing, with encouragement indicates that this leads to growth, the speed of which will vary from candidate to candidate ( Rudolph et al 2007). Gradually, the facilitator gains an impression of the learner by inquiry (what the instructor has observed informing initial comments) and then offers their frame of understanding back to the learner. This process of ‘Advocacy with Inquiry’ offers a mirror of understanding with which the candidate can concur or refute. Fanning and Gaba 2007 suggest that different levels of prior experience may affect the degree to which people can listen, process and remember information and this in turn may be reflected in the amount of facilitation required to reach learning outcomes. Three levels of instructor interaction are described:

### High level

These groups of individuals are probably experienced and are largely capable of debriefing themselves. They are reflective and the team can explore independently. The facilitator may simply need to use pauses to promote reflection, and some open ended questions, ensuring the learning outcomes are achieved.

### Intermediate

The individual and team need more help. There may be a need for more repetition of what happened, paraphrasing of answers, micro summaries to promote reflection and asking questions in different ways.

### Low level

The individual or team show little process in starting or opening up the learning conversation. The instructor has to make more observations, more reflection and more questions and the group are probably the least experienced.

### Summary

At the end of the feedback process the instructor offers one or two important suggestions which have arisen from the collaborative discussion for the lead individual and team to ‘take home’, and draws the discussion back to the learning outcomes of the simulation.

### Providing structure

Beard et al 2012 illustrated that the use a framework to deliver feedback increased the number of comments delivered to students when learning a skill. Kahn et al 2012 described how when one starts learning a skill the performance tends to be rule based and as one develops expertise there is greater fluidity and intuition (the journey towards autonomy and mastery). The mnemonic following is designed to facilitate mastery of the learning conversation.

## The Steps

In order to delineate the step wise approach to the Learning conversation the mnemonic MESSAGE may act as a framework to help instructors develop the skills of a learning conversation. There are three phases, the student’s view, the team’s view and then lastly the instructor. On resuscitation courses about five minutes is set aside from a 15 minute slot for a simulation for this process and with practise it is surprising how much useful dialogue is achieved.

### First elements concern the student view

**M**ake an opening remark

Give students the opportunity to start talking and identify their own difficulties by reflection on their action, without interruption; the candidate is less likely to bring up new issues if they are allowed to talk freely initially.

This can be encouraged by the use of certain phrases, for example:

“Take a moment to reflect on the simulation, then tell me if the simulation went according to plan?”

“What are your thoughts?”

“That looked hard”

“You looked as though you were enjoying the simulation?”

Show empathy using verbal and non verbal skills but try not to interrupt. This reaction phase differs from the narrative phase of HFS in time as the focus rests on the team leader alone in this stage. In HFS all the team will have time to explore their reaction to the simulation.

**E**xplore - Allow the candidate to explore key issues with their team as well as with the facilitator. This allows feedback to be candidate-centred.

The candidate will have their own:

• ideas of how their performance went
• concerns about their performance
• expectations of what they wish to achieve.

Work in high fidelity simulation has shown we want as far as possible for people to debrief themselves.

*These two steps explore the candidate’s viewpoint*

**S**ummarise the issues identified and reflect these back, two to three are enough.

• “so you were doubtful whether to give a second dose of adrenaline at the point?”
• “you felt you lost control of your team then too?”

This mini summary checks the facilitator has heard correctly and the body language of the candidate can be observed for receptivity.

### Second element involves group / team

**S**hare the impression these issues have made - the facilitator then shares the key issues reflecting the comments to the other team members.

“Team, you have heard this, he/she felt she lost control of the team at that point, you were the team, did you have the same impression?”

If so, what happened, what could have been done instead?

Did the team listen to the team leader?

Did team followers offer ideas/ advice/ suggestions when the team leader was struggling?

This summarising and sharing encourage the team perspective and support honest reflective and constructive opinions. This builds team followership, stressing the importance of active listening to one another. Encouraging team members to ask each other for ideas and support may help with Non Technical skills subsequently.

### Final element involves instructor

**A**dvocacy with Inquiry. Information has now been gathered and the facilitator and team now have an understanding or impression of events. The term ‘advocacy’ means to ‘speak for’ someone, so that ‘advocacy with inquiry’ is the reflection of an impression gained through questioning. If a candidate has been very self-reflective and the team supportive the instructor may simply wish to emphasise key learning and this phase is not needed. However, depending upon the maturity and bonding of the team the initial dialogue may not have explored the key educational issues until this point and Advocacy is required.

An example of how Advocacy is expressed is illustrated:

“My impression is, you were uncertain about whether or not to give the 2nd dose of adrenaline and then you and the team feel you lost your confidence. Is that right?”

By making the statement based on an observation and offering the statement back as a question it gives a candidate the opportunity to affirm or refute and correct what has been said. There is an opportunity to drive deeper into the candidate’s frames of thinking as described by Rudolf et al 2007. This is a similar process to the feedback in HFS but much shorter and some of the steps suggested by Rudolph have been removed. Argyris C 1991 suggests the facilitator is exploring the students’ beliefs or frames of thinking; exploration and critical thinking can then challenge these frames and create new learning - double loop learning. It is important that a maximum of 1-2 points are explored in order to be both useful and timely.

“ **G**ems”. Sometimes during a teaching session one hears a statement suggesting something has not been clearly understood, but the time was not appropriate to interrupt. These ‘gems’ of information need to be remembered by the facilitator and brought out in the feedback; if one person in a team did not understand something probably somebody else did not either.

**E**mphasise the key points. These often concern areas of knowledge of the algorithms or the need for the team to work harder acting as team ‘followers’ and build team leadership confidence. These three areas form the instructor view and allow opportunity to stress key learning points relating to the learning outcomes of a teaching session, avoiding repetition.

With experience this framework becomes unnecessary, dialogue will develop and flow naturally. Careful reflection and delivery of a learning conversation is important or students may find the experience difficult. The aim is to foster an open trusting relationship with students so that genuine advocacy with inquiry can occur.

## Conclusion

During simulation teaching one can promote reflection-in-action with discrete prompting but the feedback afterwards is reflection-on-action designed to reinforce performance and create double loop learning, giving the candidate increased self direction. Reflection allows students to explore their assumptions or schemata and reframe them in the light of both their experience of the simulation and the feedback. A strong evidence base for a Learning conversation has been illustrated by adapting the Calgary-Cambridge model ( Silverman et al 1997), utilising the principles of a spiral curriculum (Harden & Stamper) 1999 and challenging thinking ( Rudolph 2007, Argyris 1991) within a supportive atmosphere. The introduction of a Learning conversation on UK resuscitation courses resulted from corporate reflection and the weight of the emerging evidence and has been very successful.

Experienced instructors will maximise the learning for both individuals and the team, facilitating the team to provide its own feedback whenever possible and limiting instructor comments. A Learning conversation is an explorative dialogue which places value on the students’ ability to identify key learning ‘in context’ for them, on the team to add to the practical learning which occurs during simulation and offers a more facilitative approach which is closer to the model used in HFS. Key to its success in the authors experience is that it is vital to acknowledge remedial issues and target support in moving the candidate forward, increasing their confidence in role as a team leader. The authors hope that this style of feedback may be useful in many group teaching sessions where authentic feedback is required.

## Take Home Messages

• A Learning conversation is a means of brief, learner-focused style of feedback
• Active listening is a key skill
• The skills to develop a learning conversation can be learned and need to be practised
• A Learning conversation is an empathic and honest way to help students and faculty

## Notes On Contributors

Dr Ian Bullock PhD

CEO Royal College Physicians

An educator for Resuscitation Council for 22 years and lead educator for Resuscitation Council for 19 years.

Areas of interest:

• Clinical knowledge and skill acquisition/retention
• Simulated learning Importance of feedback to the adult learner;
• Action learning and debriefing

Dr Elizabeth M. Norris MMEd, FAcadMEd

Specialist in Medical Education

Educator for Paediatric Sub-Committee Resuscitation Council UK.

An educator for Resuscitation Council UK & ERC for 10 years.

Areas of interest:

• Curriculum design
• Non technical skills particularly management of conflict
• Barriers to learning
• Coaching and reflective practice
