# Supplementary material for: A ‘Learning conversation’ as a style of feedback
Source: MedEdPublish (2016). 2017 Sep 12;6:156. [Version 1] doi: 10.15694/mep.2017.000156 (PMC10885290; doi:10.15694/mep.2017.000156)
Supplement: Peer review history

# Reviewer report: Reviewer response for version 1

**DOI:** 10.21956/mep.19340.r28253

**Published:** 2017-09-18

**Authors:** James Fraser

**Affiliations:** 1 Griffith University

This review has been migrated. The reviewer awarded 3 stars out of 5 The importance of feedback in learning development is well understood and multiple ‘structures’ have been described. At its core feedback needs to be a dialogue or conversation and the structure described in this paper addresses a group dialogic process that is important at all levels of learning. Though I am not clear how the authors define a learning conversation. I think the mnemonic aligns with the coaching type of feedback and could be a useful tool in the clinical teaching environment. The importance of an instructor improving their practice by seeking and receiving feedback is an important point.

Reviewer Expertise:

NA

No decision status is available

# Reviewer report: Reviewer response for version 1

**DOI:** 10.21956/mep.19340.r28252

**Published:** 2017-09-16

**Authors:** Subha Ramani

**Affiliations:** 1 Harvard Medical School, Brigham and Women's Hospital

This review has been migrated. The reviewer awarded 4 stars out of 5 I appreciate the authors summarizing the older models such as Pendleton and SET-GO and proposing their own strategy for providing feedback in the simulation setting. it should be emphasized that this model is specific for simulation debrief and feedback. Besides the educational alliance approach, there is a lot of work from Canada emphasizing the sociocultural aspects of a feedback conversation- relationship, rapport, reflection as well as a coaching approach to developing action plans. These concepts are missing and perhaps do not apply. I am seeing this approach as a modified Pendleton approach for individual learners as well as the team. Breaking down the steps could be useful for many teachers in the simulation setting. The authors also cite Jenny Rudolph's work on debriefing with good judgement. I wonder whether educators might get a little confused in selecting the best model for their context if there are too many 'models' in the literature.The writing at the start did not flow smoothly or tell me the story- the problem, what has been written, what is the gap and why this model addresses that gap.But I do agree that the concept of a learning conversation would be useful to other medical educators who teach in the simulation setting. As written, I think more is needed for feedback conversations outside the simulation setting.

Reviewer Expertise:

NA

No decision status is available

# Reviewer report: Reviewer response for version 1

**DOI:** 10.21956/mep.19340.r28251

**Published:** 2017-09-14

**Authors:** Trevor Gibbs

**Affiliations:** 1 AMEE

This review has been migrated. The reviewer awarded 4 stars out of 5 Any paper that attempts to improve such an important issue as feedback is worthy of reading. I enjoyed reading this paper and believe it adds something constructive to our discussions on feedback. Like one of my co-reviewers, I was wondering about the definition of a learning conversation, although it seems to be related to taking the principles of effective feedback into a teamwork situation- a very appropriate activity.I liked the use of the mnemonic MESSAGE and believe it forms a very useful structure for all forms of feedback

Reviewer Expertise:

NA

No decision status is available

# Reviewer report: Reviewer response for version 1

**DOI:** 10.21956/mep.19340.r28250

**Published:** 2017-09-13

**Authors:** Nandalal Gunaratne

**Affiliations:** 1 Faculty of Medicine, Wayamba University of Sri Lanka

This review has been migrated. The reviewer awarded 3 stars out of 5 Interesting and informative but could have been more brief. Thank you for bringing up the difficult area of feedback on feedback.

Reviewer Expertise:

NA

No decision status is available

# Reviewer report: Reviewer response for version 1

**DOI:** 10.21956/mep.19340.r28249

**Published:** 2017-09-12

**Authors:** Ronald M Harden

**Affiliations:** 1 AMEE

This review has been migrated. The reviewer awarded 3 stars out of 5 This paper addresses an important element in medical education - FEEDBACK. - and provides an interesting discussion on aspects. It usefully draws attention to the use of learning conversations in providing feedback - an underappreciated concept. While it is worth persisting I found the paper difficult to read . There is no clear definition of what is a learning conversation. I found the description by Saunders and Gowing helpful. For me a learning conversation is where tough or negative feedback is given through a non threatening conversation eg I noticed that you did not.. .. I mention this because..what is your view? This is in contrast to a control conversation where there is a hard sell eg you did incorrectly... or the soft sell eg what do you feel you did wrong? With this in mind it was interesting to read the article

Reviewer Expertise:

NA

No decision status is available
